# Motivation to persist with internet-based cognitive behavioural treatment using blended care: a qualitative study

**DOI:** 10.1186/1471-244X-13-296

**Published:** 2013-11-07

**Authors:** Maja Wilhelmsen, Kjersti Lillevoll, Mette Bech Risør, Ragnhild Høifødt, May-Lill Johansen, Knut Waterloo, Martin Eisemann, Nils Kolstrup

**Affiliations:** 1 Department of Community Medicine, UiT The Arctic University of Norway, Tromsø, Norway; 2 Department of Psychology, UiT The Arctic University of Norway, Tromsø, Norway; 3 Department of Community Medicine, Faculty of Health Sciences, UiT The Arctic University of Norway, 9037 Tromsø, Norway

**Keywords:** Internet-based cognitive behavioural treatment, Adherence, Self-determination theory, Motivation, Depression, Primary care

## Abstract

**Background:**

The prevalence of depression is high and results in huge costs for society. Internet-based cognitive behavioural treatment (ICBT) has been suggested for use in primary care and has been shown to be more effective when combined with human support. However, non-completion rates remain a challenge. Current recommendations state that steps to improve persistence with ICBT should be determined and the impact of therapist support on persistence explored. A few earlier studies have explored motivations to persist with ICBT without face-to-face therapist support. The present study explored the motivation to persist as experienced by a group of patients who sought help in primary care and used “blended care”, i.e. ICBT supported by short face-to-face consultations.

**Methods:**

To elucidate motivation in an everyday context and the meaning of patients’ experiences we chose a phenomenological hermeneutical approach. We interviewed participants in the intervention group of a randomized controlled trial that evaluated the efficacy of an ICBT programme called MoodGYM, an eHealth intervention used to treat depression. Fourteen participants, both completers and non-completers, went through individual, semi-structured interviews after they ended their treatment.

**Results:**

Hope of recovery and a desire to gain control of one’s life were identified as intrinsic motivators. The feeling of being able to freely choose how, when and where to complete the ICBT modules was identified as an important supporting condition and satisfied the participants’ need for autonomy. Furthermore, the importance of a sense of belonging towards partners, friends or family was essential for motivation as was the ability to identify with ICBT content. Another supporting condition was the experience of connectedness when met with acknowledgement, flexibility and feedback from a qualified therapist in the face-to-face consultations.

**Conclusions:**

A key finding was that participants were motivated to persist with ICBT when their overall need for relatedness was satisfied. This was achieved through a sense of belonging towards partners, friends and family. Connectedness with the therapist and the participant’s ability to identify with the ICBT modules also gave a sense of relatedness. Improving these motivational aspects may increase patients’ persistence with ICBT.

## Background

Depression is highly prevalent, resulting in distress to patients and families and huge costs to society [1,2]. Offering early intervention benefits patients and society alike [3]. Patients tend to prefer consultations with a therapist to medication [4]. However, general practitioners (GPs) currently treat most patients with depression, and as a treatment they are widely and increasingly prescribing antidepressants [5,6]. Therefore there is a discrepancy between the treatment patients want and the treatment they get. Studies have also shown that GPs lack the necessary tools to treat mental health problems [6,7].

Internet-based cognitive behavioural treatment (ICBT) has shown promising results in treating depression [8-12]. However, Internet-based interventions have shown to yield better outcomes and greater retention when combined with therapist support [13,14]. Studies have indicated that ICBT could be widely applicable in general practice for the treatment of depression, but these studies have only a minimum of human support [4,15]. Implementing ICBT in combination with short face-to-face consultations in primary care could lead to early intervention, give increased access to cognitive behavioural treatment and improve the treatment of depression, as well as address the requests of patients for follow-up given in consultations. However, research on Internet-based interventions has shown that non-completion is a challenge [16,17]. Therefore it is recommended to find steps to improve patient persistence with Internet interventions [18] and to identify the impact of different elements of therapist support in such interventions [14,17].

According to Møller [19] health professionals often see motivation as a parameter only within the patient that can easily be measured, and this view must be challenged. She argues further that it is more interesting to explore what is motivating, rather than measuring how motivated people are. We are inspired by her conclusion that motivation to change must be seen as a complex phenomenon depending on social contexts and interpersonal relationships.

Patient motivation is an important element in persistence with any treatment programme. The Self-determination Theory (SDT) offers a broad perspective on human functioning and motivation [20-22], and says that humans naturally have intrinsic motivation, but require supportive conditions to maintain and enhance it. According to SDT, three basic psychological needs should be satisfied in order to enhance intrinsic motivation: relatedness, competence and autonomy. Relatedness includes a sense of recognition, belonging with peers, family or community and a need to feel connected to and valued by important others. Competence involves socio-contextual success with optimal challenges, feedback and freedom from demeaning evaluation. Competence will only enhance motivation if it is accompanied by a sense of autonomy [21,23].

Prochaska claims that a person goes through different stages of motivation when a change is made, and tailoring the relationship between the patient and the therapist, as well as tailoring the treatment intervention to correspond to the stage of change, can enhance the outcome [24]. To our knowledge, few studies have explored patient motivation to persist with ICBT, and those have only briefly mentioned motivation as dependent on social context [25-27]. Some barriers to ICBT completion were identified in these studies, such as lack of ability to identify with the ICBT programme applied, poor patient computer skills and the need for more therapist support [26,27]. Reported motivation to persist with ICBT includes a sense of control, an ability to identify with the ICBT programme applied, and additional support from important others [25-27]. While these studies explored some of the aspects that influence motivation to persist with ICBT without face-to-face support, the aim of this article is to explore motivation as experienced by patients using “blended care”, i.e. ICBT supported by short face-to-face consultations. These experiences will be investigated with an emphasis on everyday-life attitudes towards the ICBT programme applied, therapist support and depression. These attitudes pertain to individual patient experiences in contextual settings, rather than to motivation as a distinct quantifiable factor. The motivation to persist with ICBT is explored using the SDT as a theoretical perspective. Overall, we wish to contribute to the discussion on how to deliver improved treatment for depression to the community and to propose steps to increase persistence with ICBT.

## Methods

### Study context

From autumn 2010 until autumn 2012, a randomised controlled trial (RCT) conducted in Tromsø, Norway, offered blended care to a group of patients seeking help from their GP for mild to moderate symptoms of depression. Patients had to be between 18–65 with access to internet and those who were suicidal, psychotic, or drug abusers were excluded (for more details about the RCT see [11]). We used a Norwegian translation of an ICBT programme called MoodGYM, an eHealth intervention developed at the Australian National University. This is a free Internet-based self-help programme consisting of five interactive modules that introduce cognitive behavioural principles through online exercises. MoodGYM demonstrates the relationship between thoughts and emotions and teaches relaxation techniques. It also includes sections on managing relationships and positive behavioural activity.

Patients were asked to complete the five ICBT modules at home, and were given short, face-to-face consultations between modules with their therapist at a small clinic at the University of Tromsø. The consultations were of a supportive nature only, guided by a script consisting of three compulsory subjects: (a) symptom monitoring, (b) discussion of the topic of the last module in MoodGYM, and (c) introducing the next module and discussing patient motivation. Other issues of importance to the patient could also be discussed if there was more time. The two therapists in the study were both psychologists with limited training in CBT. To simulate the conditions in Norwegian general practice, the time spent in face-to-face consultations was 20–30 minutes, compared to 40–60 minutes in conventional cognitive behavioural treatment. The rate of non-completion was 40.4% in the intervention group of this trial. The present interview study was embedded in the RCT, trial registration: Australian New Zealand Clinical Registry ACTRN 12610000257066.

### Participants

All patients in the intervention group of the RCT were offered a debriefing session with their therapist when they ended blended care. At this time they were given written information about our qualitative research project and an informed consent form. Not all non-completers attended this session. Continuous recruitment was initially used, but was changed to strategic recruitment in order to gather a pragmatic sample number [28] of 14 consenting patients. The change from continuous to strategic recruitment was made after approximately 10 interviews to include men and women, both younger and older, and both completers and non-completers. Several non-completers refused to be interviewed. Accordingly only three non-completers were included in the present qualitative study (Table 1).

**Table 1:** Participants

**Patient**	**Age (years)**	**Male/Female**	**Completer/non-completer**
1	28	Female	Non-completer
2	26	Male	Completer
3	33	Male	Completer
4	39	Female	Completer
5	61	Female	Completer
6	41	Female	Completer
7	44	Female	Non-completer
8	26	Female	Completer
9	26	Male	Completer
10	48	Male	Completer
11	56	Female	Completer
12	36	Female	Completer
13	22	Female	Completer
14	51	Male	Non-completer

#### Interview schedule and data collection

Phenomenological hermeneutics is an analytical method developed to grasp “essential meaning” as it is lived in human experience” [29]. With a phenomenological approach lived experience is explored; how life is lived when it is taken for granted, while it contains essences of meaning. “Essential meanings” are understandable meanings of phenomena and experiences. Lived experience is explored using interviews. What is told and narrated is transformed into text which must be interpreted and a hermeneutic analysis is used. Phenomenological hermeneutics is inspired by the philosophies of Heidegger and Gadamer as well as Ricoeur [29-31]. We chose phenomenological hermeneutics as a method because it is suitable when exploring a complex phenomenon such as motivation. Our aim was to explore motivational aspects and an understanding of how the patients themselves have experienced to persist in treatment in an everyday context. By obtaining a narration of the patient’s own perspective and stories, motivation as lived experience could be explored and analysed. Also, with a phenomenological approach it was of great importance to try to set aside what we as researchers took for granted as facts and strive to listen and analyse without judgment, but concentrate on experience. As health workers ourselves, we also prepared ourselves to be open-minded, curious and set aside our medical interests. By transcribing the interviews to text and analysing the text with a hermeneutic approach we moved between understanding and explaining it [29].

Although the venue was flexible, all participants preferred to meet at a co-researcher’s office at the University of Tromsø. This office was chosen as a neutral ground and offered a slightly homey atmosphere. A pilot interview was first conducted with a test person (friend of MW) who had gone through the entire data program. Adjustments to the interview guide were made throughout the interview period. The interviewers (MW and KL) conducted semi-structured interviews designed to gather information concerning, a) a participant’s experience with the ICBT programme, b) changes in a participant’s everyday life during blended care, and c) motivational elements to persist with ICBT. Individual interviews ranged from 40–70 minutes and were recorded digitally. The interviews were performed as dialogues, and open-ended questions were used to evoke descriptions of personal experiences that reflected participants’ perceptions and feelings [31].

#### Analytical strategy and procedure

Interviews were transcribed into Nvivo 9, anonymised and then analysed in an inductive way according to the phenomenological hermeneutical method [29,30]. Findings were discussed and validated with an experienced qualitative researcher (MBR) throughout the analytical process. Inconsistencies were resolved through discussion and further reflection. Participants’ stories and reflections were analysed in a step-wise manner [29]: a) an initial reading led to the formulation of a naïve understanding; b) based on what participants said, what they talked about, and what this referred to the interview text was divided into meaning-units, then condensed and abstracted to form themes and sub-themes (Table 2). Themes were compared with the naïve understanding and the interview texts for validation; c) subsequently our findings were critically interpreted to create a comprehensive understanding and elaborated in light of existing literature and the SDT.

**Table 2:** Analysing process – example

**What participants say**		**What participants talk about**	**What participants refer to**
Meaning-unit:	Condensation:	Subtheme:	Theme:
1: I would have left the programme anyway because the modules weren’t suitable for me.	Dropped out because the modules were not suitable.	Did not identify with the programme.	Identification
2: No, to me the greatest motivation I suppose was the woman I live with. It was. And for my own part a wish to have a better everyday life […].	The greatest motivation was his wife and to have a better everyday life.	Partner is important.	Belonging/important others.

#### Ethics approval

Written informed consent was obtained from all participants. Ethical approval was given by the Regional Ethical Committee, Tromsø (2011/2163).

## Results

Relevant findings regarding the participants’ experiences of motivation were identified and are presented in two main themes combining individual patient narratives with inspiration from the SDT: a) intrinsic motivation and b) experiencing supporting conditions and persistence constraints. Another paper based on interview data from the same 14 participants focused on what they perceived as helpful and how they implemented the principles of cognitive behavioural treatment [32].

### Intrinsic motivation

#### Gaining control of one’s life

Many of our participants had struggled with symptoms of depression for many years. They were aware of their problems, but had been unable to do anything about them. When they actively took initiative or accepted treatment it was experienced as motivating. Indeed, some participants felt they had gained more control of their life by taking action:

3: But I think primarily, just getting started with something had a tremendous effect. I was actually doing something instead of sitting, waiting and feeling distressed.

5: I knew that I had a programme that I could utilise, so I did when I had time, and when I had…was in my doing mode… I felt that I wanted to take some control of the process, now I’m… […] I feel inspired to take more control.

The ability to organise their everyday life to complete the ICBT modules was also related to this feeling of gaining control. The participants’ desires to manage their own lives, expressed both directly and indirectly, indicated that this was an intrinsic motivating aspect to persist with ICBT. A transition from passive to active behaviour was a basic driving force, constituting a change from being prepared to taking action. For some it was less important what they did as long as they did something, while others emphasised that they believed in and hoped that blended care would help them.

#### Hope for recovery

Some participants had heard about cognitive behavioural treatment and had expectations that a treatment based on these principles could help them. Others had faith in blended care because their GP had recommended it. Many participants expressed their personal hope for recovery and a desire to learn more about themselves and their condition. Their hope that recovery was possible was described as motivating.

8: I wanted to get better; therefore I wanted to learn some techniques in order to function better.

13: Feeling curious [towards the programme]! Or I had hoped it would help and it has.

Participants generally described the first two ICBT modules as informative and often felt that they started a learning process, which gave them hope that it was possible to learn more. Symptoms of depression were a problem in their lives, and they wanted to continue ICBT with the hope of recovery.

### Supporting conditions and persistence constraints

#### Competence and autonomy

Learning while working on a laptop demanded very few practical adjustments from our participants, and computer skills were not an issue. The participants did not express any technical challenges of logging on or problems with manoeuvring within the ICBT programme.

*12: Yes, it went quite well. I generally spend a fair amount of time using computers anyway*.

13: For me it was not a problem working on the computer. You could do it in your own pace, relax and sit comfortably. In that way I found it to be good. The only thing was a few questions or words I didn’t understand, so you sit there alone and think… but luckily, I have Google to help me (laughter).

It is obvious that overall our participants were used to working with computers, and to using the Internet as a source of information. The mastery of technical challenges and the ability to understand the content of the ICBT programme revealed positive attitudes towards coping with it. This was conveyed together with a sense of competence.

Participants reported different experiences when the issue of time was raised, both due to long modules and competing priorities. Indeed, some modules were identified as especially time-consuming. Almost all patients expressed problems in finding time to complete the ICBT modules.

8: It was quite an effort, particularly in the beginning, when I spent a lot of time on each module.

7: It was difficult to find time for this. I’m struggling to find time to do everything. There is so much that has to be done, that most things get done half-heartedly.

Patients who were clearly taking action developed conscious strategies to overcome these constraints, such as choosing a set day to complete their modules, or using the date of the face-to-face consultation with the therapist as a deadline.

4: It was good to have a deadline [the consultation], which required me to be organised.

7: […] she [the therapist] said that this was nothing I needed to concern myself with. If it was unsuitable, I just skipped that part [module section]. It would just waste time, unnecessary time.

The flexibility to skip parts of the ICBT programme that were judged unsuitable by the participant was described as important to overcome time constraints. This flexibility was encouraged by the therapist and embraced by the participants who were strongly searching for solutions. Others were more caught up in the obstacles and were overwhelmed by time demands and long modules, but many appreciated the freedom to work at flexible hours and to select the parts of the ICBT programme they found suitable for them, which also correspond to their sense of autonomy.

#### Belonging

As mentioned above, finding time to persist with ICBT was a challenge. Participants emphasised the importance of supportive relationships, such as those with partners, friends and family who became directly involved in blended care and in the general process towards recovery. Their support and involvement became manifest and was appreciated in many ways, e.g. as encouragement to enrol in a treatment programme, to work on ICBT modules and to participate in face-to-face consultations. In this way partners, friends and family could help the participants to find the time and space to pursue their treatment.

9: No, I had my partner. I would probably have [dropped out] many times hadn’t it been for my partner [dry laughter] …she was good at pushing me… She had noticed that I felt better afterwards [consultation]. She told me that I should go [to the consultations] because I felt better after talking some… and done these tasks…

When participants allowed important others to play an active role in their treatment process, and when being open to their involvement was a positive experience, it promoted a sense of belonging, i.e. of not being alone in the process. Participants mentioned this as a motivator for moving forward in treatment.

When describing the period of treatment, the role of important others was also reported as supportive when feeling depressed in daily life. Being met with patience and that partners, friends and family were willing to make adjustments to their routines because of a participant’s limitations, was appreciated.

9: And my partner, who is quite kind [wry laughter] to me, lets me off the worst tasks. That relaxes my state of mind and in general.

14: When I haven’t been present socially and not had any social activity, questions regarding where I am and what’s wrong with me have been directed towards him [brother]. He’s been quite a helper.

These typical descriptions of caring actions revealed that the participants felt that their families or friends understood and acknowledged their problems. This pointed to the alleviating aspect of being part of social relationships during a difficult period.

Participants described that the feeling of belonging, feeling part of social relationships, directly strengthened their intrinsic hope of recovering and the motivation to engage and persist in treatment. Symptoms of depression sometimes represented a challenge to take part in social settings in a way the participant wanted. However, wanting to participate and the feeling of being welcomed by others were mutually motivating aspects. When describing what motivated participants for treatment, reasons that included their families were often expressed.

9: […] and it [engaging in treatment] was not just for my own sake, it was also for my partner’s sake.

3: Just when this happened, I became a father, and I wanted to function. I wanted to work and function. I did not want my illness to ruin the experience. Impact on the child in a negative way. The mother also needs help and support, especially just after giving birth.

Participants strove to contribute to, participate in and enjoy family life, and to interact with colleagues and friends. When this capacity was reduced it was experienced as a loss in their social life. Participants described their loss as experiencing less enjoyment and less interaction with the people they valued, which sometimes lead to isolation. Participants described this not only as a loss for them, but also for the people they were close to, revealing a confidence in feeling valued. To regain a social life was a driving force and an important motivating aspect for treatment.

Overall, the feeling of being part of a social environment, and not being alone was experienced as positive. A sense of belonging through attachment, being valued and understood, satisfied a need for relatedness and was positive for motivation. To want and be wanted socially was a driving force to engage and persist in treatment.

#### Recognition and self-identification

A positive feeling of not being alone was also experienced when participants were able to identify with and to relate to the content of the ICBT programme.

14: I found some recognisable bits there… that made it possible in a way, to connect with him or her in the various examples used. And I thought: Goodness, this is about me, right? That’s what I do!

10: Because you never know when you suddenly get an eye-opener. Wow! They [MoodGYM] hit the nail on the head again!

Patients sometimes strongly identified themselves with the fictional characters presented in the ICBT programme. This identification showed them that the problems they had, when symptoms of depression were present, were not specific to them; they were experienced by others, as well. This was perceived as essential to treatment progression and motivation.

On the other hand, participants were split on how they viewed the presentation of the ICBT content. The programme seemed superficial to some, with simplified problems, principles and strategies. It was perceived to target young people, and many expressed that the ICBT content did not correspond to their situation.

7: […] maybe it was directed towards a younger audience. Because it was a lot about exams and reading and coping with school situations. But I do understand that not everything can be right for everybody.

9: The reason I didn’t complete [all the modules completely] was because it didn’t seem right for me at the time.

The perception that the ICBT content was created to target a younger audience sometimes led to a sense of alienation. Two of the non-completers expressed difficulties with identification as the main reason for not completing the program (participant 1 and 7).

Yet, when participants gained self-identification through an ability to relate and to mirror themselves in the content, it motivated them to persist with ICBT. Discussing the ICBT programme with the therapist and important others was described as helpful in overcoming problems with the content. This strengthened the sense of identification with the content, but also the connectedness with the person in whom the participant was confiding, together satisfying the need for relatedness.

#### Connectedness and expert feedback

Participants described the face-to-face consultations as helpful and motivating. Some even expressed that they were absolutely necessary to participate in ICBT. It was through this dialogue they could reflect and develop.

3: If the consultations hadn’t been there, things would have faded somewhat.

4: […] it is in meetings with others that my thoughts fall into place. So for me it has been motivating and a very positive experience.

3: And I voiced my thoughts, and then we [patient and therapist] discussed the module. Then it was very straight forward to clear out the thoughts […]. To try to adjust the setting in the module to things that fitted me better.

Participants emphasised that the dialogue during consultations was an opportunity to put thoughts into words, rather than seeing it as a control or monitoring. It was an arena to connect, to open up, but, as mentioned earlier, was also useful to facilitate participants’ ability to identify with the ICBT content.

Connectedness was established through dialogue and strengthened when met with understanding, flexibility, acknowledgement and openness on the part of the therapist. For some, the therapist was just a person to interact with, but to others the therapist had a special expert status.

12: What was really important to me were the follow-up sessions. […] it was a person who I knew was knowledgeable and an expert on this…

Many participants wanted feedback and needed to trust the feedback given. Trust could be established when the patient was confident that the therapist was well-qualified, knew about depression and could provide therapy. This combination meant that some participants felt they were getting expert feedback. When asked, many expressed that their GP could not function as a therapist in the same manner, giving reasons such as lack of time and lack of competence in cognitive behavioural therapy.

Time constraints made the consultation stressful to some participants, both completers and non-completers alike. All three non-completers wanted to discuss their problems in more depth and wanted a more individualised approach in the face-to-face consultations as in traditional therapy. One of the non-completers was offered this and chose it instead of continuing with blended ICBT (participant 14).

1: The consultations could have been longer and more in-depth… about other things and how I functioned otherwise in life.

For some patients, short consultations were a barrier to open up and satisfy their need for connectedness. However, to summarise, all patients emphasised the importance of the dialogue with the therapist. Working with ICBT alone was not preferable.

## Discussion

A key finding was that our participants were motivated to persist with ICBT when their need for relatedness was satisfied. The SDT claims that the psychological needs for relatedness, competence and autonomy must be satisfied to enhance intrinsic motivation [21,23]. All three were identified to be influential in our study, especially relatedness in terms of recognition, belonging and connectedness. Our findings show how motivation to persist with ICBT was not only an internal individual process, but also a social process where interactions with others were essential. Unique to this study were the findings exploring what aspects of the interaction with the therapist were important for motivation. These aspects were identified as connectedness through acknowledgement, trustworthy feedback and encouragement of flexibility. Our findings are in line with Johansson’s findings of a better outcome when there is a more human involvement during follow-up [13] as some of our participants expressed face-to-face consultations to be absolutely necessary to participate in ICBT. Major barriers to ICBT persistence and completion, such as lack of support, lack of computer skills and lack of self-identification were not expressed as clear in our data as in the study by Donkin et al., which was found in an older population without therapeutic consultations [25]. This could imply that the short face-to-face consultations in our study helped increase acceptability and enhanced supporting conditions. The way questions were asked during the interview could also explain the difference in findings. Santana has shown that use of the Internet to assess health problems is rising [33]. The fact that our population was younger (see Table 1) and therefore more skilled in using the Internet to obtain information about health issues may be a reason for not experiencing computer skills as a barrier. A reason could also be that possibly MoodGYM is a more user-friendly programme yielding these findings.

However, not being able to identify with the content of the ICBT programme was identified as a barrier in our findings, especially with the non-completers and has been found in earlier studies [25,27]. The SDT claims that if ideas and values are not shared, relatedness is hindered, resulting in a feeling of alienation [21]. Our findings indicated that social relationships with the therapist and important others could facilitate the process of self-identification, and thereby enhance relatedness and motivation. On the other hand, depression per se may compromise the ability to be self-determined. According to the SDT, the more self-determined patients are, the more likely they are to engage in activities that reflect their own values and interests, and such activities are more likely to satisfy psychological needs [20]. Kaylai has found that being depressed is to be “unhomelike”, i.e. a state of alienation [34]. Alienation in the SDT is the opposite of relatedness, and can undermine self-motivation [21]. This barrier is inevitable. However in follow-up of patients suffering from depression the therapist can help to establish supportive conditions to satisfy the need for relatedness and thus hopefully enhance motivation. As mentioned in our findings, the relationship with the therapist, but also with partners, friends and family were important. According to the SDT, feelings of identification, belonging and connectedness satisfy the need for relatedness [21]. In earlier studies, exploring ICBT, social relations were mentioned to be important by enhancing and maintaining motivation [19,21,35]. This supports the assumption that relatedness is important for motivation.

Many participants pointed out that a feeling of taking action and a need for feedback from an expert therapist, who in our study was a psychologist, was of great importance. Time constraints were identified as a problem to connect with the therapist. Especially the non-completers expressed a need for a more traditional therapy with more time and in-depth dialogue around their problems. However, most of our participants experienced trust and acknowledgement in their consultations, despite their short length. According to Prochaska, tailoring the therapeutic relationship and treatment intervention to the patient’s stage of change can enhance the outcome, specifically the percentage of patients completing therapy, and the ultimate success of treatment. While in the stage of action, patients need the advice of an expert [24]. In our findings, patients often told stories that indicated that they were in a stage of action when motivated to persist in treatment. The fact that the therapist was a psychologist, an expert, was experienced to strengthen this motivation. GPs experiences with short consultations indicate that they feel they are able to give useful treatment for depression, despite time pressure [5]. On the other hand, patients reported that time constraints in consultations with GPs undermine their capacity to benefit from the consultation. The presence of other patients in the waiting room just made matters worse as it was experienced as more stressful that other patients waited for the GP, and made it more difficult to open up and connect [5,36]. In our study the therapist was never disturbed, and the waiting room was almost always empty. Possibly this setting fostered relatedness despite the short consultations. If blended care is implemented in general practice, patients may feel stress due to appointment time constraints and may fail to feel connected with the GP. It is also uncertain whether a GP or other ICBT provider in primary care can fill the shoes of being an expert during the process of change. Undertaking training in the delivering of blended ICBT may provide competence for such GPs.

### Strengths and limitations

A methodological strength with this study was the use of in-depth interviews. Participants themselves were thus able to elaborate on their experiences with ICBT. Depending on the situation, people in social interactions inhabit different role characters and statuses [37]. In our study, both interviewers were first of all researchers, but they were also health professionals. The second author was a therapist in the RCT in which this study is situated, but never interviewed her own patients. We chose to be open about our double status, but did not inform our participants about her status as a therapist. We made it clear that our intention was not to defend the treatment, but to better understand the patients' thoughts and experiences. The intention was to open an honest and trusting dialogue. It was a strength that the experienced third author (MBR) continuously gave feedback on our interviews to ensure that quality and adequate depth was achieved.

Participants entering RCTs are a selected sample [38] and may therefore be more motivated. One strength of our study was that all participants had initially contacted their GP for their problems, which may reduce the selection bias. A limitation was that only three of our 14 participants were non-completers. Although non-completers were invited to be interviewed, many refused and thus could not be included in our study. Therefore our findings can only be seen as a partial description of the full range of patients. However, our main aim in this article was to explore the aspects of motivation to start and persist with ICBT, and accordingly a majority of completers was acceptable.

## Conclusions

By analysing the interviews of our 14 participants, we attempted to understand motivation as experienced when using ICBT with short intermittent consultations. Intrinsic motivators such as gaining control of one’s life and a personal hope for recovery were identified. A key result was that patients were motivated to persist with ICBT when their need for relatedness was satisfied. This was achieved through feelings of self-identification with the ICBT programme and an experience of belonging and being valued by partners, friends and family and connectedness with the therapist.

### Implications for research and practice

Enhancing motivation may be a step to improving therapeutic persistence. However, further research is necessary. We propose four steps an ICBT deliverer in primary care should have in mind in the encounter with patients with depression to increase motivation.

First, communicating hope by educating patients about the effectiveness of ICBT and the good prognosis of depression. Second, encouraging patients to enlist the support of important others in their process towards recovery. Third, communicating that the ICBT deliverer has competence and can give qualified feedback. Fourth, focusing on acknowledgement, flexibility and understanding in the meeting with the patient. This may increase a feeling of connectedness and autonomy.

Future research is needed to explore in more depth whether blended ICBT can represent a worthwhile treatment for depression in primary care and be added as an additional tool in a GP's armoury.

## Abbreviations

ICBT: Internet-based Cognitive Behavioural Treatment; GP: General Practice; RCT: Randomised Controlled Trial; SDT: Self-determination Theory.

## Competing interests

The authors declare that they have no competing interests.

## Authors’ contributions

The research-group; MW, KL, RH, ME, KW, MR and NK met on a regular basis to depict the design of the study, discuss and make all necessary decisions during the entire research process, accessing all relevant papers (e.g. interview-guide, consent-form, coding). MW and KL were responsible for data-collection, transcribing data and analysing data. MW wrote the first draft of the manuscript. MR and KL contributed continuously with comments and discussion to data-collection, coding, analysis and writing of this paper. MJ contributed to both to analysis and in the writing of the manuscript. All authors commented on drafts of this paper. All authors read and approved the final manuscript.

## Pre-publication history

The pre-publication history for this paper can be accessed here:

http://www.biomedcentral.com/1471-244X/13/296/prepub
